# Anti-diabetic therapies on dental implant success in diabetes mellitus: a comprehensive review

**DOI:** 10.3389/fphar.2024.1506437

**Published:** 2024-12-11

**Authors:** Hamzeh Ghorbani, Arsen Minasyan, Delaram Ansari, Parvin Ghorbani, David A. Wood, Rozi Yeremyan, Simin Ghorbani, Natali Minasian

**Affiliations:** ^1^ Young Researchers and Elite Club, Ahvaz Branch, Islamic Azad University, Ahvaz, Iran; ^2^ Faculty of General Medicine, University of Traditional Medicine of Armenia (UTMA), Yerevan, Armenia; ^3^ Department of Dentistry, University of Traditional Medicine of Armenia (UTMA), Yerevan, Armenia; ^4^ Department of Cardiology, Faculty of Medicine, Ahvaz Jundishapur University of Medical Sciences, Ahvaz, Iran; ^5^ DWA Energy Limited, Lincoln, United Kingdom; ^6^ Department of Nursing and Midwifery, Faculty of Nursing, Ahvaz Jundishapur University of Medical Sciences, Ahvaz, Iran; ^7^ Faculty of General Medicine, Yaroslavl State Medical University, Yaroslavl, Russia

**Keywords:** hyperglycemia, dental implants, anti-diabetic medications, type 1 diabetes mellitus (T1DM), type 2 diabetes mellitus (T2DM), implant stability

## Abstract

**Background and Objective:**

Dental implant therapy faces challenges in patients with Type 1 and Type 2 Diabetes Mellitus (T1DM and T2DM) due to adverse effects on bone metabolism and immune response. Despite advancements, diabetic patients face higher risks of peri-implantitis and compromised osseointegration. This review assesses the impact of anti-diabetic medications on implant outcomes, offering insights to bridge the gap between animal studies and clinical practice. By evaluating pharmacotherapeutic strategies in preclinical models, this review guides future research designs to improve implant success rates in diabetic individuals.

**Method:**

A comprehensive literature review identified 21 animal studies examining the impact of anti-diabetic medications on dental and bone implants. These studies explored diabetes models, medication regimens, and designs to assess outcomes related to bone metabolism, osseointegration, and peri-implant tissue responses. The findings are systematically summarized, highlighting the scope, design, and procedures of each study. An example includes placing a dental implant in the molar region of a mouse, providing insight into preclinical approaches.

**Results:**

Twenty-one animal studies, primarily using rodents, investigate various anti-diabetic medications on dental and bone implants. Interventions include insulin, aminoguanidine, voglibose, sitagliptin, exenatide, and metformin, analyzing outcomes like bone-implant contact (BIC), bone volume (BV), and counter-torque values in T1DM and T2DM models. The impacts of these medications on implant osseointegration under diabetic conditions are detailed, with their benefits and shortcomings assessed.

**Discussion:**

The findings and challenges of existing animal studies on diabetes mellitus (DM) and implant osseointegration are presented. Despite T2DM prevalence, research primarily focuses on T1DM models due to easier experimental practicalities, limiting applicability. Inconsistent protocols in studies compromise reliability regarding anti-diabetic treatments’ effectiveness on osseointegration. Standardized methodologies and long-term assessments of local drug delivery alongside systemic anti-DM treatments are crucial to manage DM-related complications in implant dentistry.

**Conclusion:**

Insulin administration in short-term T1DM animal studies enhances implant osseointegration. However, the efficacy of non-insulin medications remains inconclusive. Rigorous experimental designs are needed to address inconsistencies and assess long-term impacts. Larger-sized (e.g., porcine) animal studies across various intraoral implant scenarios are required. Future research should focus on enhancing clinical applicability and improving implant stability in evolving conditions.

## Highlights

• Improved dental implant therapy in diabetic patients is needed.
• Impact of anti-diabetic drugs on dental implants: animal studies versus clinical outcomes.
• Insulin impacts identified in bone mineral content in Type 1 Diabetes Mellitus (T1DM).
• Disparities exist between animal studies and clinical implant outcomes.
• Critical knowledge gap exists in dental implant performance with T2DM conditions.

## 1 Introduction

Type 2 Diabetes Mellitus (T2DM) is prevalent among the elderly population, as reported by the World Health Organization (WHO) in 2016, with a significant increase observed in recent decades (Who, 2016). Hyperglycemia associated with T2DM contributes to various oral complications, including heightened susceptibility to periodontal diseases, compromised oral immunity, and delayed oral wound healing (Kocher et al., 2018). Consequently, individuals with T2DM are at an elevated risk of tooth loss (Jimenez et al., 2012).

Dental implant treatment is an established practice for replacing missing teeth offering a practical alternative to dental bridges and removable dentures (Preoteasa et al., 2015). Despite initial concerns, research has investigated peri-implant complications, such as marginal bone loss and peri-implantitis, in diabetic patients to ascertain their suitability for dental implant treatment (Gómez-Moreno et al., 2015; Aldahlawi et al., 2021). The prevalence of diabetes mellitus (DM) globally is on the rise and affecting younger as well as older age groups, largely attributed to increasing rates of obesity and more sedentary lifestyles. Recent studies indicate a substantial number of individuals are now affected by DM, and those numbers are projected to rise further by 2050 (Ong et al., 2023).

Diabetes constitutes a significant risk factor for periodontitis, a condition closely linked to tooth loss (Nascimento et al., 2018; Lau et al., 2022; Naseer et al., 2024). Several hormones play pivotal roles in regulating bone metabolism (Niwczyk et al., 2023), which in turn significantly affects dental implant success (Niwczyk et al., 2023). Parathyroid hormone (PTH) promotes bone formation by enhancing osteoblast activity and inhibiting osteoclast activity, which can support bone remodeling around implants. Insulin, crucial for glucose regulation, also plays a key role in bone metabolism, promoting osteoblast differentiation and bone formation. Estrogen and testosterone, both of which influence bone density (Mills et al., 2021), are especially relevant in postmenopausal women and men with osteoporosis. Testosterone deficiency is linked to increased risk of osteoporosis, potentially affecting implant success. Adiponectin, typically associated with fat tissue, has been shown to stimulate osteoblast activity and suppress osteoclast formation, which may enhance bone healing. Also, oxytocin, known for its role in labor, promotes bone formation and reduces bone resorption. Despite the potential of these hormones to enhance osseointegration, diabetes-induced hormonal imbalances can complicate their beneficial effects on bone healing (Tunheim et al., 2023). The collective effects of the mentioned hormones have the potential to improve dental implant outcomes, though more research is required to optimize their clinical application (Tunheim et al., 2023). Consequently, individuals with diabetes are more susceptible to tooth loss and edentulism, irrespective of their glycemic control status (Vu et al., 2022). Recent research has identified the intricate relationship between hyperglycemia and implant failure in individuals with T2DM. While systemic complications of T2DM are well-established, the impact on bone metabolism and, consequently, implant outcomes have emerged as a significant concern. Building upon previous findings, contemporary investigations have delved into the molecular mechanisms underlying impaired bone healing and compromised osseointegration in the context of elevated glucose levels.

Recent studies have expanded upon the results of animal studies to explore human clinical data, providing valuable insights into the translational relevance of earlier experimental findings (Sábado-Bundó et al., 2019; Ayele et al., 2023). In particular, advanced imaging techniques such as micro-CT scanning combined with histomorphometry analyses have offered detailed assessments of bone-implant interfaces in diabetic patients (Ding et al., 2019). These analyses have revealed distinct morphological alterations and reduced bone volume fractions surrounding implants in individuals with poorly controlled T2DM (Xiang et al., 2020). These results are indicative of impaired osseointegration.

The role of chronic inflammation and oxidative stress in exacerbating implant complications within hyperglycemic environments has garnered considerable attention (Corduas et al., 2020). The utilization of dental implants among individuals with T2DM remains a contentious issue due to the potential detrimental impact of hyperglycemia on osseointegration. T2DM is associated with heightened inflammatory responses to oral biofilms, potentially exacerbating predisposition to gingivitis (Sundar, 2018). Current evidence is insufficient to support comparable outcomes of implant therapy between patients with and without DM. Kopman et al. (2005), using rat models, demonstrated the inhibitory effects of diabetes on osseointegration, evidenced by reduced bone-to-implant contact (BIC) (Kopman et al., 2005).

**FIGURE 1:**
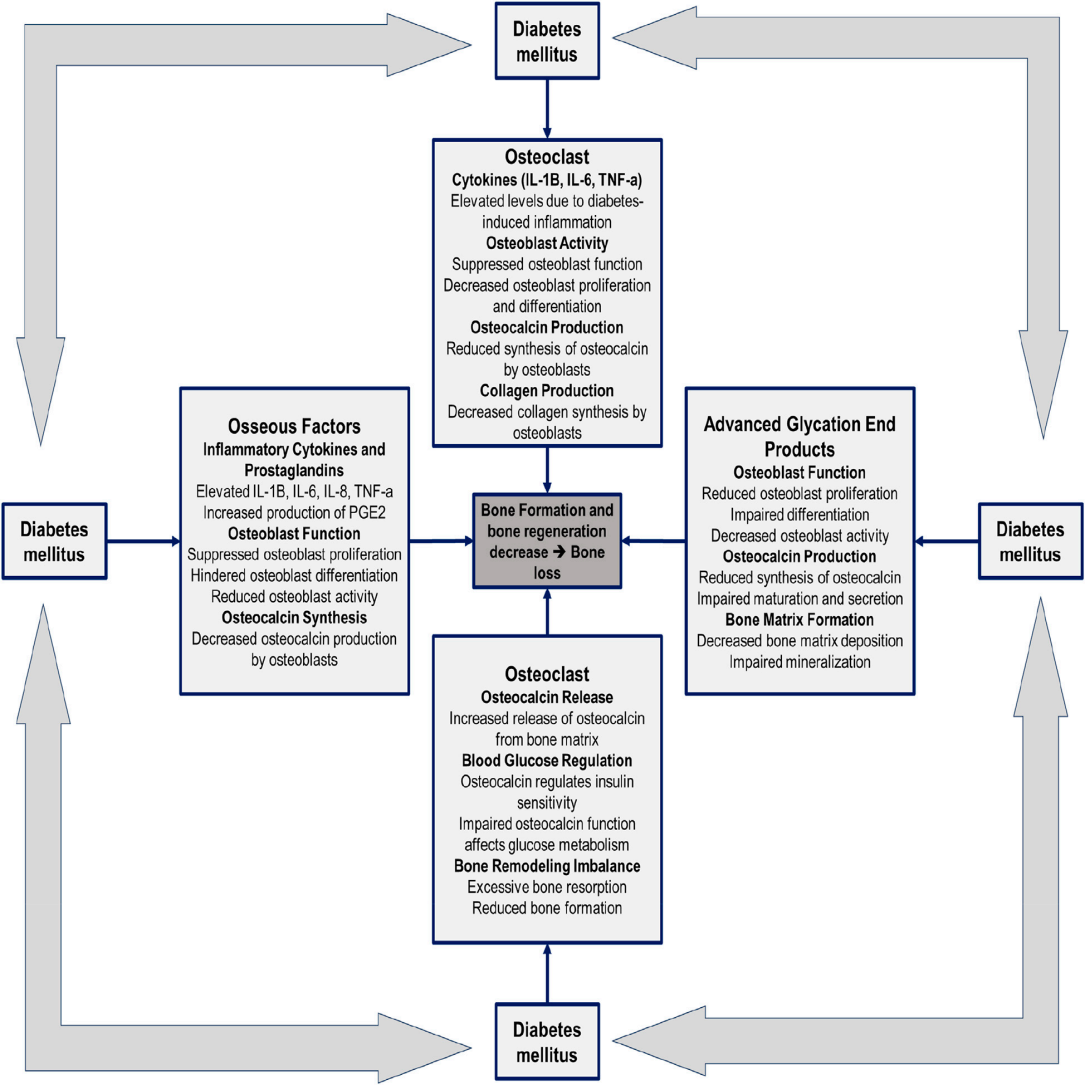
Factors influencing the pathogenesis of insufficient bone formation and bone loss resulting from diabetes mellitus.

Human research indicates increased alveolar bone loss in diabetic patients (Tabassum, 2022), potentially due to elevated secretion of proinflammatory cytokines including interleukin (IL)-1b, IL-6, and tumor necrosis factor-alpha (TNF-a) in serum and gingival fluid. These cytokines are also attributed to accelerated age receptor (RAGE) interactions. Heightened expression of proinflammatory cytokines within bone tissues suggests an intrinsic inflammatory response in the bones of diabetes suffers, potentially enhancing Osteoclastogenesis and bone resorption. Figure 1 show the pathogenesis of the insufficient bone formation and bone loss due to the DM. Consequently, tailored and clinically proven strategies for implant placement and management in diabetic patients are required. Ongoing research is focusing on the development of novel biomaterials and pharmacotherapeutic interventions to modulate inflammatory responses to dental implants. Bridging basic science and clinical practice holds promise for improving long-term dental implant success rates in individuals with T2DM. Table 1 summarizes the diagnostic challenges and hurdles in diagnosis, management, complications, monitoring, and patient education associated with T2DM and dental implants.

**TABLE 1:** Diagnostic challenges and implant considerations in T2DM management.

Category	Diagnostic challenges/Problems related to T2DM	Challenges with dental implants
Diagnosis	✓Insidious onset, often asymptomatic	✓Increased risk of peri-implantitis due to compromised immune response and delayed wound healing
	✓Overlapping symptoms with other conditions	✓Difficulty in distinguishing between implant failure and diabetic complications
	✓Variability in diagnostic criteria and guidelines	✓Poor glycemic control leading to implant failure
Management	✓Complexity in treatment regimens	✓Increased risk of infection and delayed healing
	✓Need for individualized care plans	✓Potential interactions between anti-diabetic medications and antibiotics used in implant surgery
	✓Adherence issues with medication and lifestyle changes	✓Higher prevalence of periodontal disease, requiring meticulous oral hygiene
Complications	✓Increased risk of cardiovascular disease, neuropathy, and retinopathy	✓Dental implant failure due to bone loss and poor osseointegration
	✓Hypoglycemia and hyperglycemia episodes	✓Risk of peri-implant mucositis and peri-implantitis
	✓Delayed wound healing	✓Challenges in anesthesia management due to altered pain perception
Monitoring	✓Regular monitoring of blood glucose levels	✓Frequent follow-ups to monitor implant stability and peri-implant tissues
	✓Screening for diabetic complications	✓Diagnostic imaging to assess bone density and implant positioning
	✓Collaboration between dental and medical professionals	✓Integration of dental implant care into overall diabetes management
Patient Education	✓Importance of self-management and lifestyle modifications	✓Educating patients about oral hygiene practices and implant maintenance
	✓Recognition of warning signs and symptoms	✓Signs of implant failure and when to seek dental care
	✓Understanding the impact of diabetes on oral health	✓Potential impact of diabetes on implant success and longevity

This review collates and assesses information on the types of drugs used for anti-diabetic management in the context of dental procedures, including dental implants. The bulk of the existing research on anti-diabetic drugs is related to animal studies, with relatively few based on human studies and clinical trials. This discrepancy represents a research gap that justifies more focused human studies. It is well established that findings from animal studies often serve as a precursor to human trials, rendering this avenue of investigation invaluable to researchers and clinicians engaged in this field of study. Hence, it is important to understand the existing body of knowledge gained from animal studies regarding anti-diabetic therapies on dental implants to assist in the design and execution of future human studies.

## 2 Method

**FIGURE 2:**
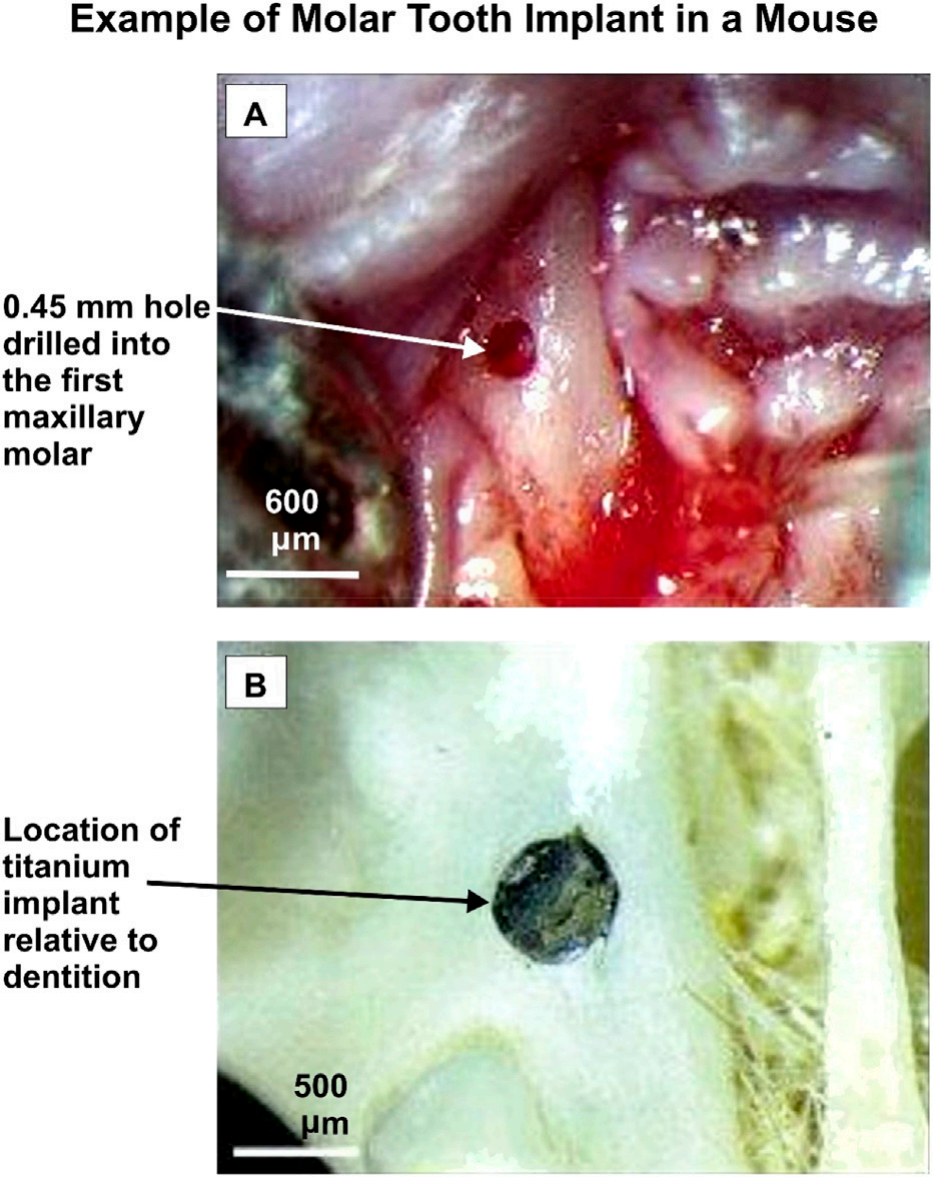
An example of a molar tooth implant in a mouse. Adapted from Mouraret et al. (2014).

A comprehensive literature review identified twenty-one published studies focusing on animal models to explore the effects of anti-diabetic medications on dental and bone implants. These studies investigated various experimental designs, including the induction of diabetes using agents like Streptozocin or alloxan and the administration of medications such as insulin and metformin. The research analyzed outcomes related to bone metabolism, osseointegration, and peri-implant tissue responses. Figure 2 illustrates an example of dental implant placement in a mouse model. Table 2 provides a detailed summary of the study scopes, methodologies, and results. This approach highlights the preclinical evidence base for understanding the interplay between diabetes, its treatments, and implant success.

**TABLE 2:** Summary of animal studies on anti-diabetic medications following dental and bone implants.

No.	Year	Authors	Reference	Description
01	1999	Fiorellini et al	Fiorellini et al. (1999)	Ten male 5-week-old Sprague-Dawley rats were divided into two groups of five, with each group receiving implant placement. DM was induced in all rats via a single intraperitoneal injection of 70 mg/kg Streptozocin before the implantation procedure. The rats were monitored post-injection, and diabetes was confirmed on day 2. Insulin therapy was initiated to manage T1DM.
02	2001	Matsubara et al	Matsubara et al. (2001)	Male Wistar rats, each 12 weeks old, were divided into three groups with 12 rats per group, totaling 36 rats. These rats were used to model T1DM, which was induced using a single dose of 60 mg/kg Streptozocin administered before implant placement. Insulin was used as the medication for managing diabetes in these rats. The timing of diabetes induction and subsequent implant placement was carefully controlled, with diabetes being induced prior to the placement of any implants
03	2003	Siqueira et al	Siqueira et al. (2003)	43 male Wistar rats, aged 12 weeks, were divided into control (n = 17) and diabetic (n = 18) groups, with 8 rats receiving insulin treatment. Diabetes was induced via intraperitoneal alloxan injection at a dosage of 42 mg/kg. Implants were placed before diabetes induction, and the effects were assessed 10 days post-implantation. This setup offers a comprehensive model to investigate the impact of diabetes and insulin treatment on various physiological parameters in rats
04	2003	Margonar et al	Margonar et al. (2003)	New Zealand rabbits, 20 weeks old and all female, were divided into three groups, each consisting of nine individuals, totaling twenty-seven. DM was induced through intraperitoneal alloxan injection at a dosage of 115 mg/kg before implant placement. The type of DM induced was T1DM, necessitating insulin medication. The onset of diabetes occurred on the second day post-induction, marking the commencement of the study
05	2005	Kopman et al	Kopman et al. (2005)	Male Sprague-Dawley rats were used as the animal model to investigate the effects of Type 1 T1DM induced by aminoguanidine. The induction of diabetes was achieved using a single dose of 70 mg/kg Streptozocin administered before implant placement, with diabetes management starting on the day of the implant placement. The experiment involved a total of 32 rats, divided into groups of 8, each receiving implants to analyze the outcomes under diabetic conditions. The specific age of the rats was not provided in the study
06	2005	Kwon et al	Kwon et al. (2005)	Sprague-Dawley rats, 4 weeks old males, were used in groups of four, totaling 32 individuals. Diabetes was induced using Streptozocin at a dosage of 70 mg/kg, with implants placed 28 days prior to induction. The type of diabetes modeled was T1DM, managed with insulin medication. The induction of diabetes occurred 28 days after implant placement
07	2006	McCracken et al	McCracken et al. (2006)	Sprague-Dawley rats, a total of 152 animals were divided into diabetic and healthy groups, with 60 rats induced with T1DM through 60 mg/kg Streptozocin injection before implant placement. Among the diabetic cohort, 32 rats received insulin treatment, but the specific details regarding the sex, and age of the rats was not specified. The induction of diabetes preceded the implant placement, indicating a controlled experimental design, with ongoing insulin therapy administered to maintain diabetic conditions throughout the study
08	2009	de Morais et al	De Morais et al. (2009)	Forty male Wistar rats, aged 16 weeks, were divided into four groups with ten rats each. T1DM was induced using 40 mg/kg Streptozocin, administered 2 months *after* implant placement. On day 2 post-induction, all rats exhibited signs of diabetes. They serve as a model for understanding the effects of insulin treatment and diabetes management
09	2011	Guimarães et al	Guimaraes et al. (2011)	Wistar rats, 20 weeks of age, were divided into groups with six rats in each group, totaling 36 subjects. The type of diabetes induced was T1DM, using aminoguanidine as the medication. Diabetes was induced through an intraperitoneal injection of alloxan at a dosage of 84 mg/kg before implant placement. The timing of diabetes induction began on the day the implants were placed. The specific number of implants per rat was not specified
10	2011	Wang et al	Wang et al. (2011)	Goto-Kakizaki (GK) rats were utilized, along with 10 control Sprague-Dawley rats, to investigate T2DM induction and management. The GK rats were characterized by spontaneous T2DM and underwent implant placement with slow-release insulin coating, while Sprague-Dawley rats served as controls. The specific details regarding the sex, age, and number of implants were not provided. However, the induction of T2DM in the GK rats was attributed to their genetically modified nature. This research offers insights into the efficacy of slow-release insulin implants in managing T2DM in spontaneously diabetic animals
11	2012	Han et al	Han et al. (2012)	Wistar rats, specifically male, were utilized with six rats per group, totaling 48 rats. Diabetes was induced in the rats through the administration of 80 mg/kg Streptozocin prior to implant placement. The type of diabetes modeled was T1DM, managed with insulin. Notably, a slow-release insulin-coated implant was used during the implant placement process, ensuring sustained medication delivery
12	2013	Aiala et al	Aiala et al. (2013)	48 Male Wistar rats aged 28 weeks, were divided into groups of 8 to investigate the effects of DM on implant outcomes. The induction of T1DM was achieved using 70 mg/kg of Streptozocin administered with aminoguanidine before implant placement. The onset of diabetes was synchronized to begin on the day of implant placement to assess its immediate impact on the implants
13	2013	de Molon et al	De Molon et al. (2013)	80 male Wistar rats, 16 weeks old, were divided into four groups of 20 rats. These rats were induced with T1DM through an injection of 40 mg/kg of Streptozocin. Following the induction of diabetes, the implant placements were performed on day 2 of diabetes management, with the rat’s receiving insulin as part of their treatment
14	2014	Inouye et al	Inouye et al. (2014)	36 male GK rats at 12 weeks of age, were divided into four groups of 12 rats. These rats were models of T2DM induced spontaneously due to genetic modification. They were implanted while in a diabetic state, with ongoing metformin treatment as the medication regimen. This approach allowed for the examination of metformin’s effects on already established T2DM in GK rats, offering insights into potential therapeutic interventions for managing the condition in its advanced stages
15	2014	Hashiguchi et al	Hashiguchi et al. (2014)	20 male GK rats, ten per group, were utilized as a model for Type 2 T2DM. These rats were genetically modified to spontaneously develop T2DM, The diabetic rats were induced with dental implants with ongoing Voglibose treatment as part of their medication regimen. This approach offers insights into the progression and treatment of T2DM in a controlled experimental setting
16	2015	Liu et al	Liu et al. (2015)	33 male Zucker diabetic fatty (ZDF) rats,12 weeks old, were divided into three groups of 11 rats, with each rat induced with two dental implants. These rats were induced with T2DM using exenatide, and they had also been genetically modified, spontaneous T2DM. The induction of diabetes occurred in spontaneously diabetic animals, and the treatment involved ongoing subcutaneous injection
17	2015	Zhou et al	Zhou et al. (2015)	33 male Zucker diabetic fatty (ZDF) rats,12 weeks old, were divided into three groups of 11 rats, with each rat induced with two dental implants. The rats were induced with T2DM using exenatide, a medication administered through ongoing subcutaneous injections. Notably, these rats were genetically modified to develop spontaneous T2DM, with implant placement occurring in already diabetic animals, providing a valuable model for studying the progression and treatment of diabetes
18	2017	Ribiero Serrão et al	Ribeiro Serrão et al. (2017)	30 Male Wistar rats, aged 12 weeks, were divided into groups of 10, with each group undergoing implant placement. The induction of T2DM was achieved through a combination of 10% fructose diet and Streptozocin administration at 40 mg/kg. Diabetes was induced 2 weeks after implant placement, resulting in a total of 30 spontaneously diabetic animals across the groups. Metformin was administered as medication, with each group receiving the same dosage. This experimental setup allows for a comprehensive investigation into the effects of T2DM and medication on the implanted rats within a controlled environment
19	2019	Bautista et al	Bautista et al. (2019)	32 male Wistar rats aged 16 weeks were divided into groups of 8 for the investigation. The research focused on T1DM induced by administering 40 mg/kg Streptozocin, with sitagliptin used as the medication. The induction of diabetes occurred before the placement of implants, with the treatment starting the day after the implants were placed
20	2020	Yamazaki et al	Yamazaki et al. (2020)	36 male Wistar rats, aged 5 weeks, were divided into four groups of 12 rats. Type 1 diabetes was induced using 50 mg/kg Streptozocin, administered 5 weeks after implant placement. The onset of diabetes occurred on Day 3 post-induction, with insulin being utilized as the medication regimen
21	2021	Zhang et al	Zhang et al. (2021)	30 male Sprague–Dawley rats, aged 11 weeks, were divided into five groups of 6 rats, and used to investigate the effects of T1DM on implant outcomes. Diabetes was induced using a 30 mg/kg dose of Streptozocin to create a model of T1DM, and insulin was administered as the treatment medication. The induction of diabetes occurred before the implant placement, ensuring the condition was established prior to the intervention

Table 2 describes, in summary, the scopes, designs and procedures of those studies.

## 3 Result

The 21 studies described incorporated a variety of pharmacological interventions for diabetes management, including:

(A) Insulin as the treatment drug
(B) Non-Insulin treatment drugs:
  • Aminoguanidine
  • Voglibose
  • Sitagliptin
  • Exenatide
  • Metformin

**FIGURE 3:**
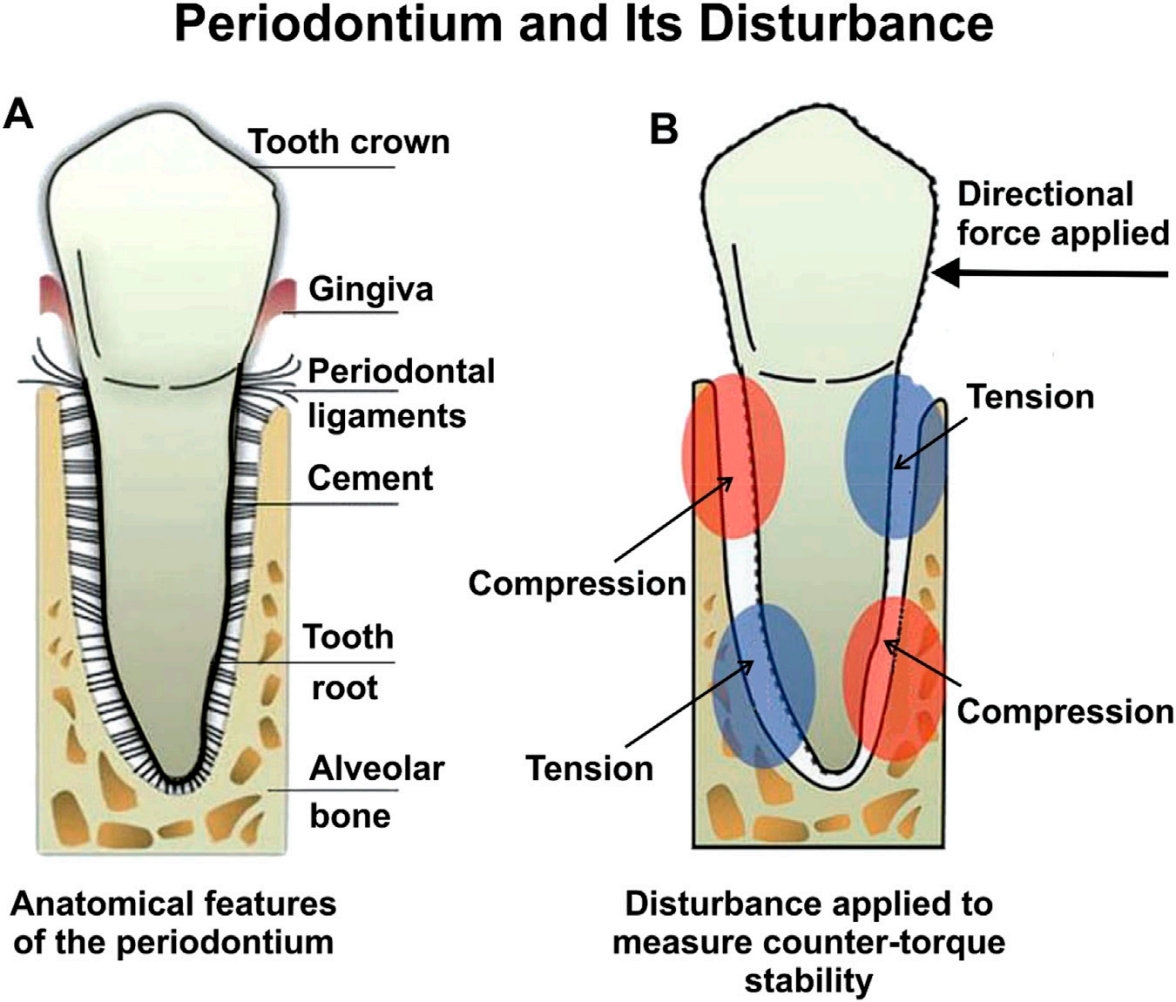
**(A)** Periodontium features, and **(B)** Periodontium disturbance when measuring counter-torque stability. Adapted from d’Apuzzo et al. (2017).

Four of these studies concentrated on T1DM and explored how effective non-insulin medications could be as treatments (Kopman et al., 2005; Guimaraes et al., 2011; Aiala et al., 2013; Bautista et al., 2019). One study focused on T2DM and investigated the impact of insulin treatment on the progression of osseointegration (Wang et al., 2011). Key measures of interest included bone-implant contact (BIC), bone volume (BV), and counter-torque values (N/cm), which served as indicators of the progress of implant osseointegration. Figure 3 provides a schematic of the periodontium and the disturbance impacts related to counter-torque measurements. The periodontium, as depicted in Figure 3, plays a critical role in the stability and success of dental implants, especially in individuals with diabetes. Figure 3A illustrates the normal anatomy of the periodontium, including key components such as the tooth crown, gingiva, periodontal ligaments, cementum, tooth root, and alveolar bone. These structures work cohesively to provide structural support and respond to mechanical forces. Figure 3B demonstrates how directional forces exert tension and compression on the periodontium, with distinct red and blue regions indicating these stress zones. This mechanical response is vital in assessing counter-torque stability—a key metric for evaluating the success of dental implants. In the context of diabetes, the periodontium is particularly vulnerable due to several physiological disruptions. Elevated blood glucose levels impair wound healing, reducing the ability of the periodontium to recover from surgical procedures such as implant placement. Additionally, diabetes-induced vascular damage limits blood flow to the gingiva and supporting tissues, weakening their structural integrity and making them more prone to infection. Compounding this is the immunosuppressive effect of diabetes, which diminishes the body’s ability to combat infections, including peri-implantitis—a condition marked by inflammation and infection around the implant site (Schwarz et al., 2018). These factors collectively increase the risk of delayed healing, compromised osseointegration, and implant failure in diabetic patients. The mechanical stresses shown in Figure 3 further highlight the challenges of achieving successful implant integration in compromised tissues, emphasizing the need for meticulous management of diabetes and tailored dental care to enhance implant outcomes.

The role of pharmacological interventions in managing diabetes is pivotal for improving dental implant success in individuals with Type 1 and Type 2 Diabetes Mellitus (T1DM and T2DM) (Rokaya et al., 2020). Different classes of anti-diabetic drugs, including insulin, GLP-1 receptor agonists, DPP-4 inhibitors, biguanides, and alpha-glucosidase inhibitors, have shown varying effects on glycemic control, bone metabolism, and implant osseointegration in both preclinical and clinical studies (Rokaya et al., 2020). While insulin remains critical for its direct impact on osseointegration and bone healing, medications like metformin and exenatide have demonstrated potential benefits by enhancing osteoblast activity and reducing inflammation. Conversely, some medications, such as bisphosphonates, may negatively affect implant outcomes by impairing bone healing (Kalaitzoglou et al., 2019). Table 3 summarizes these medications and their specific effects on dental implant success.

**TABLE 3:** Summary of anti-diabetic medications and their effects on dental implant success.

Medication	Mechanism of action	Effects on dental implant success
Insulin	Regulates glucose metabolism and enhances bone formation	Improves osseointegration, increases bone-to-implant contact (BIC), and supports implant stability
Metformin	Reduces gluconeogenesis and enhances insulin sensitivity	Promotes osteoblast activity, increases bone density, but inconsistent long-term effects on BIC.
Exenatide (GLP-1 RA)	Stimulates insulin release and reduces inflammation	Enhances osteoblast attachment and bone density, improving peri-implant healing
Sitagliptin (DPP-4 Inhibitor)	Prevents incretin degradation to stabilize glucose levels	Limited impact on bone parameters; hyperglycemia persists in T1DM models
Voglibose	Inhibits alpha-glucosidase to delay carbohydrate absorption	Minimal improvements in BIC and osseointegration; hyperglycemia affects efficacy
Aminoguanidine	Reduces oxidative stress and advanced glycation end-products	Enhances implant stability and BIC in animal models, but concerns over human application

Table 4 provides detailed information regarding the placement of implants, the specific medications used, their ability to control blood sugar levels, and the corresponding outcomes of implants for each of the mentioned studies.

**TABLE 4:** Animal studies on anti-diabetic medications and bone implants: key outcomes of interest in implant osseointegration.

No.	Year	Authors	Reference	Key outcomes on implant osseointegration
01	1999	Fiorellini et al	Fiorellini et al. (1999)	Systemic insulin improved bone formation and BIC% in diabetic rats, achieving levels comparable to healthy controls
02	2001	Matsubara et al	Matsubara et al. (2001)	Insulin normalized glucose levels and increased BIC% in diabetic animals, demonstrating improved bone volume and integration
03	2003	Siqueira et al	Siqueira et al. (2003)	Insulin enhanced BIC% in diabetic rats, indicating that glucose control is critical for successful osseointegration
04	2003	Margonar et al	Margonar et al. (2003)	Insulin treatment significantly improved torque strength, reflecting better implant stability in diabetic rabbits
05	2005	Kopman et al	Kopman et al. (2005)	Aminoguanidine increased BIC% and improved osseointegration compared to untreated diabetic controls, despite persistent hyperglycemia
06	2005	Kwon et al	Kwon et al. (2005)	Systemic insulin effectively enhanced BIC% and bone-implant integration in diabetic animals over a long-term observation period
07	2006	McCracken et al	McCracken et al. (2006)	Insulin improved bone volume in diabetic rats, though levels remained lower compared to healthy animals
08	2009	de Morais et al	De Morais et al. (2009)	Insulin treatment prevented peri-implant bone loss and increased bone density in diabetic conditions
09	2011	Guimarães et al	Guimaraes et al. (2011)	Aminoguanidine enhanced implant stability as measured by counter-torque, improving outcomes in diabetic rats
10	2011	Wang et al	Wang et al. (2011)	Locally delivered insulin significantly increased BIC% and improved osseointegration in diabetic animals
11	2012	Han et al	Han et al. (2012)	Local insulin delivery enhanced mechanical stability and BIC%, highlighting its efficacy in diabetic conditions
12	2013	Aiala et al	Aiala et al. (2013)	Aminoguanidine improved BIC% and implant stability, although diabetic conditions continued to affect overall outcomes
13	2013	de Molon et al	De Molon et al. (2013)	Systemic insulin reduced glucose levels and significantly enhanced BIC%, bone area, and implant stability in diabetic conditions
14	2014	Inouye et al	Inouye et al. (2014)	Metformin improved BIC% and peri-implant bone healing, but hyperglycemia persisted in treated diabetic animals
15	2014	Hashiguchi et al	Hashiguchi et al. (2014)	Voglibose increased BIC%, but statistical significance in implant stability compared to untreated diabetic controls was not achieved
16	2015	Liu et al	Liu et al. (2015)	Exenatide improved bone density, reduced inflammation, and enhanced integrin expression, promoting bone healing in diabetic rats
17	2015	Zhou et al	Zhou et al. (2015)	Exenatide promoted peri-implant bone formation, improving bone healing and integration in diabetic conditions
18	2017	Serrão et al	Ribeiro Serrão et al. (2017)	Metformin increased BIC% and osteoprotective factors, reducing bone loss in diabetic animals
19	2019	Bautista et al	Bautista et al. (2019)	Sitagliptin improved bone area occupancy but failed to mitigate the significant bone loss in diabetic conditions
20	2020	Yamazaki et al	Yamazaki et al. (2020)	Systemic insulin improved biomechanical integrity and prevented bone loss, achieving BIC% comparable to healthy controls
21	2021	Zhang et al	Zhang et al. (2021)	Insulin enhanced implant stability and BIC%, although diabetic bone integrity was not fully restored

The studies reviewed in Tables 3, 4 investigate the impacts of various drugs on bone integration in animal models of DM. Aminoguanidine, metformin, sitagliptin, and insulin were among the drugs scrutinized for their effects on osseointegration. Aminoguanidine demonstrated potential in enhancing BIC% and mechanical stability in diabetic animals, despite persistent hyperglycemia. Conversely, sitagliptin, which stimulates insulin secretion, did not improve bone parameters due to ongoing hyperglycemia and the destruction of pancreatic β-cells in the diabetic models studied. Insulin, whether administered systemically or locally, consistently showed benefits in enhancing BIC%, peri-implant bone density, and mechanical retention in both T1DM and T2DM animal models, underscoring its important role, not only in glycemic control, but also in bone metabolism and remodeling. These findings highlight the complex interplay between diabetes, anti-diabetic medications, and bone health, suggesting that while some drugs may improve bone outcomes, sustained hyperglycemia can hinder their efficacy (Table 5). Despite the potential of anti-diabetic medications, their efficacy in promoting bone healing and osseointegration is often limited by persistent hyperglycemia. This challenge is underscored by contrasting results across animal studies, where some medications, such as insulin, show promise in controlling blood glucose and promoting bone healing, while others, like sitagliptin, fail to overcome the detrimental effects of sustained hyperglycemia. These outcomes highlight the critical need for more effective glycemic control alongside anti-diabetic treatment to optimize implant outcomes.

**TABLE 5:** Impact of diabetes medications on dental and bone implant results in animal models of T1DM and T2DM.

Drug	Studies	Findings	DM type	Administration site	Key points
Aminoguanidine	Kopman et al. (2005), Guimaraes et al. (2011), Aiala et al. (2013)	Increased BIC and counter-torque values; hyperglycemia maintained	T1DM	Intraperitoneal; Local membrane	Does not affect glucose metabolism
Sitagliptin	Hashiguchi et al. (2014)	No reduction in blood glucose or influence on bone parameters; hyperglycemia maintained	T1DM	Oral	Ineffective in T1DM due to β-cell destruction
Insulin	De Morais et al. (2009), De Molon et al. (2013), Matsubara et al. (2001), Zhang et al. (2021), Kwon et al. (2005), Fiorellini et al. (1999), Siqueira et al. (2003), Margonar et al. (2003), McCracken et al. (2006), Han et al. (2012), Bautista et al. (2019), Yamazaki et al. (2020)	Improved BIC, bone density, and mechanical retention; some conflicting results on bone volume	T1DM	Systemic; Local sustained-release vehicles	Critical for osseointegration and bone metabolism
Metformin	Ribeiro Serrão et al. (2017), Inouye et al. (2014)	Initial improvement in BIC lost over time; increased OPG expression	T2DM	Systemic	Possible molecular benefits, but inconsistent BIC results
Voglibose	Hashiguchi et al. (2014)	No improvement in BIC or torque strength; hyperglycemia maintained	T2DM	Systemic	Ineffective in reversing hyperglycemia’s effects on bone
Exenatide	Zhou et al. (2015), Liu et al. (2015)	Elevated ALP, increased integrin α5β1 and fibronectin expression, improved bone density	T2DM	Systemic	Enhances osseointegration via osteoblast attachment
Insulin	Wang et al. (2011)	Increased BIC in insulin-coated implants; hyperglycemia level unspecified	T2DM	Local (implant–bone interface)	Promotes new bone formation locally

## 4 Discussion

It is commonly recognized that well-conducted animal studies are crucial for producing strong molecular and cellular evidence that can guide improvements in clinical practice. A scoping review highlighted a predominant focus on T1DM models despite T2DM being more prevalent in humans. The likely reason for this bias is the cost-effectiveness and ease of disease induction for T1DM compared to T2DM. The research involved various animal strains and widely differing methodologies for diabetes induction, anti-diabetic medication administration, disease timing, and location of implant placement. One of the most significant parameters in animals is blood glucose levels, which can directly affect both T1DM and T2DM models. Throughout the studies, animals often remained hyperglycemic, mirroring poorly controlled diabetes in humans. However, inconsistent glycemic control, deviations from experimental protocols, and a lack of standardization have undermined the reliability and conclusiveness of findings concerning the effects of anti-diabetic treatments on implant osseointegration. Moreover, assessments of potential bias in the studies revealed significant shortcomings in accounting for experimental variables, reinforcing the critical need for researchers to address blinding, randomization, disease severity heterogeneity, husbandry practices, and reporting standards to ensure robust study design and reliable outcomes.

Peri-implantitis, characterized by inflammation and progressive bone loss, further exacerbates challenges in diabetic patients. Factors such as microbial biofilms, impaired immune responses, and reduced vascularization at implant sites significantly affect outcomes (Rokaya et al., 2020). The risk of peri-implantitis, characterized by inflammation and bone loss around implants, is heightened in diabetic patients due to several factors. Elevated glucose levels impair neutrophil function, a crucial component of the immune system, thereby increasing the risk of infection and inflammatory responses at implant sites. Additionally, the increased levels of matrix metalloproteinases (MMPs), particularly MMP-8, further accelerate bone degradation, making it difficult for implants to integrate properly. These biochemical and immune responses collectively exacerbate the risk of peri-implantitis, underscoring the importance of managing hyperglycemia to reduce infection risks and improve implant success. Systemic and local factors that influence bone metabolism, such as vitamin D deficiency or elevated pro-inflammatory cytokines, also exacerbate disease progression (Rokaya et al., 2020).

Certain medications may inadvertently promote peri-implantitis. For example, bisphosphonates, while beneficial for osteoporosis, can impair bone healing and increase the risk of osteonecrosis in dental implant sites. Conversely, drugs like metformin and GLP-1 receptor agonists, which enhance bone metabolism and reduce inflammation, may mitigate these risks. Local drug delivery systems, such as insulin or chlorhexidine-coated implants, have shown promise in reducing peri-implant inflammation and improving outcomes, although their long-term benefits remain unclear (Rokaya et al., 2020).

Most of the studies considered involved short durations (4–16 weeks), and all but one study (Margonar et al., 2003) used rodents. The preference for rodents as experimental subjects is associated with their accelerated bone metabolism and turnover rates. This tends to reduce experimental expenses and reduce animal care requirements (Omar et al., 2020). However, the relatively small size of rodents limits the type of surgery and implant placement that can be performed; constraining it to approximately 1 × 2 mm dimensions (Blanc-Sylvestre et al., 2021). Consequently, rodent studies involve limitations when considering analogous clinical scenarios in humans (Prabhakar, 2012; Mohd et al., 2022a; Mohd et al., 2022b). For example, rodent implant studies commonly involve the tibia or femur (larger bones) rather than maxillary/mandibular dental extraction sockets. Oral-cavity implants face a different set of issues to leg bones, including, including bacterial plaque, masticatory forces, exposure to food particles and microorganisms, which are constant sterility threats to intraoral implants (Blanc-Sylvestre et al., 2021). Hence, questions exist regarding the extrapolating from rodent test results to likely human outcomes. Porcine experimental subjects (e.g., Göttingen minipigs) can provide a better scale of animal subject for oral implants with greater similarity to humans regarding bone regeneration and metabolism. In porcine experiments it is possible to conduct multiple jawbone implants facilitating a broader spectrum of biomarker and genetic bone-impact research model (Musskopf et al., 2022; Coelho et al., 2018).

The animal studies reviewed used various anti-diabetic medications including biguanides, alpha-glucosidase inhibitors, GLP-1 analogs, DPP-4 inhibitors, and insulin. Aminoguanidine acts by scavenging free radicals and blocking AGE formation, thus reducing oxidative damage and complications associated with AGEs (Guimaraes et al., 2011). Sitagliptin, a DPP-4 inhibitor, enhances glycemic control by preventing the degradation of incretins like GLP-1, thereby stimulating insulin release and stabilizing post-meal blood sugar levels. Metformin reduces gluconeogenesis and intestinal glucose absorption while enhancing peripheral insulin sensitivity (Jiating et al., 2019). Voglibose, an alpha-glucosidase inhibitor, competitively blocks glucose absorption after meals (Chen et al., 2017). Exenatide, an agonist of the GLP-1 receptor, stimulates insulin release, suppresses glucagon activity, and delays gastric emptying. Despite these mechanisms, most non-insulin medications did not achieve sufficient control of blood glucose levels or improve osseointegration in the animal studies reviewed (Kopman et al., 2005; Aiala et al., 2013; Inouye et al., 2014; Hashiguchi et al., 2014; Liu et al., 2015; Zhou et al., 2015; Ribeiro Serrão et al., 2017). Sitagliptin’s effectiveness was questioned in rat models of T1DM, raising doubts about its utility in research (Kilkenny et al., 2010). Aminoguanidine demonstrated promise in enhancing healing around implants in animals with T1DM (Kopman et al., 2005; Guimaraes et al., 2011; Aiala et al., 2013), but concerns over significant reported side effects in humans have clouded its clinical application (Bolton et al., 2004). Insulin, vital for managing T1DM, has shown positive effects on glucose regulation, promoting osseointegration, and reversing bone changes compared to other medications (Kwon et al., 2005). There are gaps in research regarding the impact of systemic insulin in T2DM animal models and its comparison with outcomes in T1DM, highlighting the need for future studies to consider diabetes pathophysiology and drug mechanisms to evaluate effectiveness, safety, and influence on implant osseointegration.

Three of the studies reviewed involved local drug delivery directly at the implant sites in attempts to enhance osseointegration (Wang et al., 2011; Han et al., 2012; Aiala et al., 2013). Although initial outcomes showed improved BIC and counter-torque values, the benefits were temporary, as systemic hyperglycemia persisted in the animals. Unfortunately, these short-duration test results provide no indication of the longer-lasting impacts of the treatments involved on osseointegration. It is therefore important to conduct further longer-term tests to more fully assess the post-drug release impacts on the progress of osseointegration, as ongoing bone responses may change over time following initial implant placement. The existing evidence suggests that bone resorption, reduced BIC, and ultimately implant failures are potential long-term effects of uncontrolled DM (Kwon et al., 2005; De Molon et al., 2013; Yamazaki et al., 2020). Closer scrutiny is required of the potential long-term human clinical benefits of implant-site-specific anti-DM drug delivery as either an alternative to, or a complement to, the more usual systemic anti-diabetic treatments.

## 5 Conclusion

Diabetes Mellitus (DM), encompassing both Type 1 (T1DM) and Type 2 (T2DM), poses substantial challenges to dental implant therapy due to its detrimental effects on bone metabolism and immune function. Despite advancements in implant technology, diabetic patients face heightened risks of peri-implantitis and compromised osseointegration. This review has synthesized findings from 21 animal studies investigating the impact of anti-diabetic medications on dental and bone implants, aiming to provide insight regarding the gap between preclinical research and clinical practice. The pharmacotherapeutic approaches of the various diabetes models evaluated, including the use of insulin and non-insulin agents like aminoguanidine, Voglibose, sitagliptin, exenatide, and metformin, reveal conflicting short-term outcomes. Dental and bone implant studies typically record and assess bone-implant contact (BIC), bone volume (BV), and counter-torque values. The results of the available short-term studies in T1DM models demonstrate the beneficial effects of insulin on enhancing BIC and implant retention. However, the effectiveness of non-insulin medications applied to implant subjects afflicted with T1DM and T2DM remains inconclusive.

The disparity between animal model findings and clinical applicability underscores critical methodological gaps, including inconsistent glycemic control, disparate study durations, and variable drug dosages and delivery methods. The interplay between bone metabolism, systemic health, and local peri-implant conditions underscores the complexity of managing dental implants in diabetic patients. Further research is warranted to optimize therapeutic strategies, standardize study designs, and evaluate the long-term effects of anti-diabetic and adjunctive therapies on peri-implant health. These discrepancies limit the translation of animal study results to human diabetic conditions. These shortcomings necessitate standardized experimental protocols and the consideration of local (implant-site-specific) drug delivery alongside systemic treatments to address diabetes-related complexities in implant dentistry more effectively. Moving forward, more comprehensive evaluations are required in larger-sized animal (e.g., porcine) models and intraoral implant settings. Such studies are required to substantiate or refute the findings published to date from mainly rodent-based, leg-bone studies. It is also important for future studies to focus on the potential long-term enhancement of implant stability amidst evolving diabetic scenarios. Consequently, there is a need for future research endeavors to prioritize extended preclinical investigations to assess long-term implant performance in humans developing T1DM and T2DM at different ages, thereby guiding optimized therapeutic strategies tailored to improve implant outcomes in diverse diabetic populations.

## Nomenclature

BAFO: Bone Area Fraction Occupancy
BIC: Bone-To-Implant Contact
DM: Diabetes Mellitus
GK: Goto-Kakizaki
OPG: Osteoprotegerin
RANKL: Receptor Activator of Nuclear Factor Kappa-B Ligand
T1DM: Type 1 Diabetes Mellitus
T2DM: Type 2 Diabetes Mellitus
TNF-a: Tumor Necrosis Factor-Alpha
WHO: World Health Organization
ZDF: Zucker Diabetic Fatty
